# Comments on the New Edition (12th) of Harris' Principles and Practice of Dentistry

**Published:** 1889-06

**Authors:** 


### COMMENTS ON THE NEW EDITION (12TH) OF HARRIS' PRINCIPLES AND PRACTICE OF DENTISTRY.

The Principles and Practice of Dental Surgery, by Chapin A. Harris, M. D., D. D. S. Revised and edited by Ferdinand J. S. Gorgas, A. M., M. D., D. D.S., author of Dental Medicine, Professor Principles of Dental Surgery and Mechanism, University of Maryland Dental Department. P. Blakeston, Son & Co., Philadelphia, publishers. Cloth, $7.00, leather $8.00.

Why is it not enough simply to mention this standard work on dentistry, because it is not the same that it was, but has been rebuilt, enlarged and refurnished. This encyclopedia has been translated into many languages.

America has consumed many editions. We now have the twelfth edition with an additional three hundred and twenty-six pages more than its immediate predecessor. Three hundred and eighty-two new illustrations have been added, all of which adds value to the already invaluable book. New systems in practice have been added; in fact, practice and theory are brought down to date. For sale by Holliday Bros.—*Southern Dental Journal.*

---

It is a very happy thought to keep this grand monument to a grand man in frequent repair. There have been many books on dentistry written since our Chapin Harris died, but not one of them will live longer in the hearts of the profession, old and young, than this standard for students and stand-by for practitioners. Harris could hardly have asked a better posthumous tribute than the revision, for the twelfth time since 1840, of his early love in dental literature; and it would have been no bad idea, had many more of our modern authors studied this work as a model of composition, as well as a compendium of practice, before issuing their own productions. Not only has this work "reached every civilized country, but has been translated into several languages." Hardly a chapter but has been revised, and about 226 new pages, and 382 new illustrations have been added. The late Prof. P. H. Austen supervised the revision of the tenth edition, and Prof. Gorgas, of Baltimore, gave a great impetus to the fresh popularity of the work when he revised the eleventh. By his labors on this last edition, he has not only perpetuated the great reputation of Harris, but merits for himself another niche for another statue. The dental student may learn all he may ever need of dental anatomy and physiology, which occupies 161 pages with about 80 illustrations. In dental pathology and therapeutics the work covers dentition, diseases of the mucous membrane, diseases of the gum, tumors of the mouth and jaws, calcic deposits of the teeth, the fluids of the mouth, diseases of the pulp, alveolar processes, etc. In dental surgery are comprised irregularities, treatment of caries, extraction, anæsthetics, etc. The natural prejudice many of us entertain against the profuse display of manufacturers' catalogues in text books, will be provoked here again, but the text is so well prepared, and there is such a faithful attempt made to enlighten the student, that one feels like overlooking it. Dental mechanics cover the whole range of mechanism, introducing crown and bridge work, almost all taken from published sources. This department is a complete treatise in itself. It is impossible to say too much for the editor as well as publishers of this useful work. Every dentist should add it to his library. Every student should make it his own.—*Dominion Dental Journal.*

---

This reliable work is so well known to the profession that it is not necessary to go into details regarding its contents, but we will, however, note some of the changes that have been made in this new edition. Additions have been made to almost every chapter and new matter added to such an extent that this new twelfth edition contains some two hundred and twenty-six pages more than the eleventh edition, notwithstanding the omissions deemed necessary. Three hundred and eighty-two illustrations have been added and several new systems not before published, in works of this character—appear in this volume. Everything is brought into conformity with the latest and best principles and methods of the profession.

That the work has reached every civilized country and been translated into several languages is evidence of its value as a text book. Including, as it does, Anatomy, Physiology, Pathology, Therapeutics, Dental Surgery and Mechanics, it covers a large field and the reader is enabled to glean the latest and best of information from its pages. The portion devoted to Dental Surgery covers 333 pages and that on Dental Mechanics 490 pages. It is extremely practical and can be truly said to contain more valuable information regarding the principles and practice of dentistrv than any other one volume ever published. We heartily recommend it to all practitioners and students who have need of such a work; and who has not?—*Ohio Journal of Dental Science.*

---

Twelve editions, running through nearly a half-century of uniform adoption as the principal text-book in all dental educational institutions, indicates the recognized value of this initial work of the father of modern dentistry. The present volume, of twelve hundred and twelve pages and index, and more than one thousand illustrations, comprises the later phases of dental progress in principles and practice, and is creditable alike to editor and publisher. Neither the student, the practitioner, nor the teacher is fully equipped without the possession of this volume.—*Dental Cosmos.*

